# Prevention of upper limb symptoms and signs of nerve afflictions in computer operators: The effect of intervention by stretching

**DOI:** 10.1186/1745-6673-3-1

**Published:** 2008-01-07

**Authors:** Jorgen R Jepsen, Gert Thomsen

**Affiliations:** 1 Department of Occupational Medicine, Sydvestjysk Sygehus, Østergade 81-83, DK-6700 Esbjerg, Denmark

## Abstract

**Background:**

In a previous study of computer operators we have demonstrated the relation of upper limb pain to individual and patterns of neurological findings (reduced function of muscles, sensory deviations from normal and mechanical allodynia of nerve trunks). The identified patterns were in accordance with neural afflictions at three specific locations (brachial plexus at chord level, posterior interosseous and median nerve on elbow level). We have introduced an intervention program aiming to mobilize nerves at these locations and tested its efficacy.

**Methods:**

125 and 59, respectively, computer operators in two divisions of an engineering consultancy company were invited to answer a questionnaire on upper limb symptoms and to undergo a blinded neurological examination. Participants in one division were subsequently instructed to participate in an upper limb stretching course at least three times during workdays in a six month period. Subjects from the other division served as controls. At the end of the intervention both groups were invited to a second identical evaluation by questionnaire and physical examination. Symptoms and findings were studied in the right upper limb. Perceived changes of pain were recorded and individual and patterns of physical findings assessed for both groups at baseline and at follow-up. In subjects with no or minimal preceding pain we additionally studied the relation of incident pain to the summarized findings for parameters contained in the definition of nerve affliction at the three locations.

**Results:**

Summarized pain was significantly reduced in the intervention group but unchanged in controls. After the intervention, fewer neurological abnormalities in accordance with nerve affliction were recorded for the whole material but no conclusion could be drawn regarding the relation to the intervention of this reduction. Incident pain correlated to findings in accordance with the three locations of nerve affliction.

**Conclusion:**

A six month course of stretching seems to reduce upper limb symptoms in computer operators but we could not demonstrate an influence on neurological physical findings in this sample. The relation of incident symptoms to identified neurological patterns provides additional support to the construct validity of the employed neurological examination.

## Background

Neck-shoulder-arm pain is frequent among computer workers but there is a controversy with regard to the character of disorders responsible for these symptoms and their prevention remains a challenge. However, in heavily exposed symptomatic computer operators it is possible to identify neurological abnormalities including selective muscle weakness, deviation from normal of sensibility, and mechanical allodynia of nerve trunks. The occurrence of physical findings in distinct neurological patterns suggests the involvement of the brachial plexus at chord level (located infraclavicularly behind the pectoralis minor muscle), and of the posterior interosseous and median nerves at elbow level [1]. These patterns were identified by a neurological examination which has been previously shown to be reproducible and reflecting symptoms [2-4].

Applied to a sample of computer operators with few and minor symptoms, a similar neurological examination has demonstrated the presence and relation to symptoms of specific patterns of abnormalities [5]. While this study did not intend to determine the relation of upper limb symptoms to computer work, the findings are concurrent with hypotheses of external causation of work-related upper limb nerve-afflictions [6,7] including the relation to office work [8].

Researchers seem to share the view that computer use (hours per day or week) is related to upper limb morbidity [9]. However, those involved in computer intensive work, e.g. computer aided design, are reluctant to accept half-time jobs and other preventive options likewise appear insufficient. A recent Cochrane review has concluded that there is limited evidence for the efficacy of exercises and breaks and that the benefit of ergonomic interventions has not been clearly demonstrated [10]. While education in office ergonomics has resulted in less pain and discomfort in some studies [11-13] others have failed to demonstrate this effect [14]. The effect of work environment improvements seems to be superior when combined with changes in work techniques [15]. One study has suggested the preventive role of forearm support [16] while others found no effect of postural interventions [17].

The failure of ergonomically designed workstations to satisfactorily prevent adverse musculoskeletal health effects has been attributed to their inability to correct for a major contribution of constrained posture. To address this factor computer operators have been recommended physical exercises many of which, however, have been regarded as conspicuous and potentially embarrassing to perform, as disruptive of work routines, as posing health hazards by exacerbating the biomechanical stress in computer work, or as contraindicated in subjects with certain health problems [18]. The limited evidence for the effectiveness of exercises may be due to their content which constituted strengthening and endurance rather than stretching [10]. One study has shown that frequent short breaks from computer work improve productivity and well-being when the breaks integrate with task demands – especially when combined with stretching exercises [19]. In another study recovery from upper limb and neck complaints was promoted by regular breaks but there was no additional effects of physical exercises [20]. In a review of the efficacy of stretching for prevention of injury related to exercise (sports) no conclusions could be drawn due to the paucity, heterogeneity and poor quality of studies [21]. Isolated stretching exercises have not been studied in computer operators but were rated as beneficial by ultrasonography staff with musculoskeletal complaints [22]. The static components in ultrasound examinations may be comparable to that of computer work but the forces involved are higher and the variability of upper limb posture probably greater.

A recent systematic review of the effect of interventions among computer users found that our ability to draw conclusions about ergonomic interventions including the effect of rest brakes and exercises was limited by the small number of good quality studies [23].

Our clinical observations have indicated that upper limb symptoms and physical findings may still develop in computer workers in spite of attempts to optimize ergonomics and work organization, e.g. by reducing computer workload through addressing deadlines and overtime. The current insufficiency of effective preventive measures suggests the need for a broader scope.

Physical findings in computer operators [5] suggest that at specific anatomic locations with narrow passages nerve trunks may be compressed, tethered or fixed by surrounding structures. Accordingly, a rational preventive approach would aim to maintain nerve-mobility at these locations. This may be accomplished by influencing gradients of tissue pressure in order to improve capillary blood flow and venous return in nerves [24,25] and by re-establishing muscle balance (e.g., through strengthening of specific muscles and stretching of their antagonists) [7].

These considerations and prior encouraging experiences [19,22] influenced our decision to study if stretching exercises aiming to mobilize the nerves at specific locations can reduce upper limb symptoms in computer operators. The demonstration of a beneficial effect of such targeted stretching would contribute to the prevention of upper limb pain in computer operators and also provide a further validation of the previously presented diagnostic approach [2-4].

We have aimed to test the value of such an intervention.

## Methods

### Design

The study was a controlled interventional trial and involved one company with divisions in several cities countrywide. The work tasks in each division were comparable. Allocation to the intervention group or control group was based on geography with the intervention department located in the city of Esbjerg and the control department geographically separated in Aarhus.

Before and after the intervention, data were collected by questionnaires and physical examinations.

### Material

**Figure 1:**
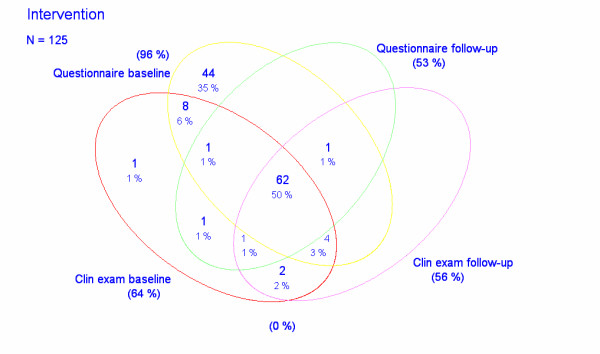
Wenn diagram illustrating the studied samples of intervention subjects.

**Figure 2:**
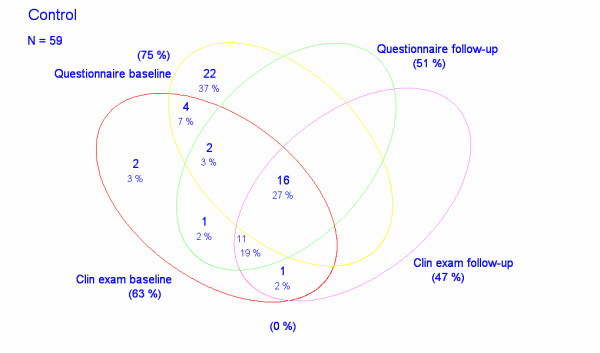
Wenn diagram illustrating the studied samples of control subjects.

The study base consisted of 125 and 59 computer operators, respectively, in two divisions of a Danish engineering company Rambøll A/S situated in Esbjerg and Aarhus, respectively (Figures 1, 2). All participants were employed as engineers or technical assistants. They were selected for being exposed to graphical computer work for more than 20% of their total working time or having experienced upper limb symptoms within the last 12 months.

The study complied with the Helsinki declaration. It was approved by the local Ethics Committee (2487A-03) and signed informed consent was obtained from all participants.

### Questionnaire

The questionnaires were based on the Nordic Questionnaire [26] and designed for electronic completion and submission. The posed questions included perceived pain during the last three months. Answers were scored on a VAS-scale 0 ("no pain") – 9 ("intolerable pain") for each of three regions (shoulder, elbow, and hand/wrist) on both sides. The questionnaires employed at baseline and at follow-up were identical except for additional questions in the latter on the extent to which the respondent had participated in the intervention and whether the symptoms in each region had changed. The latter was reported on a 5 point scale from "much worse" to "much better".

### Physical examination

Selected neurological parameters which were included in a formerly presented detailed examination protocol [2,3] were semi-quantifiable assessed (Table 1). The following parameters were examined bilaterally:

**Table 1:** Quantification of the neurological qualities examined

**Examined quality**	**Interpretation**
Manual isometric muscle testing in individual muscles [2]	5 Contraction against powerful resistance/normal power = 0 4+ Contraction against gravity and strong resistance = 1 4 Contraction against gravity and moderate resistance = 2 4- Contraction against gravity and slight resistance = 3
	
Mechanosensitivity with slight pressure along nerve trunks [3]	No soreness = 0 Mild mechanical allodynia = 1 Moderate mechanical allodynia = 2 Severe mechanical allodynia = 3
	
Sensibility examined by needle prick (algesia) and tuning fork 256 Hz (vibratory threshold [3])	Normal sensibility = 0 Reduced/changed sensibility = 1 Severely reduced/changed sensibility = 2

• 11 individual muscles (Table 2) were manually tested simultaneously on the two sides in order to reveal any discrepancy in between the right and left side. Aiming to stabilize the limb, minimize discomfort and ensure a biomechanical optimal positioning during testing of a specific muscle while disfavouring the influence of others, specific postures have been carefully defined for each muscle. Up to three reiterations of each test were performed in order to identify abnormal fatigue. The intent was to assess the peak function as well as the ability of the individual to hold the force at a constant level during testing thus containing a component of endurance. The level of function of each muscle was graded between 0 and 5 with subdivision of grade 4 into 4-, 4, and 4+ [2,27] (Table 1).

**Table 2:** Reported change in symptoms at follow-up for responders to the second questionnaire analyzed by Wilcoxon rank-sum (Mann-Whitney) test

	**Intervention subjects. N = 66**			**Control subjects. N = 30**			
**Region**	**Worse or much worse**	**Unchanged**	**Better or much better**	**Worse or much worse**	**Unchanged**	**Better or much better**	**P**

Shoulder	3	47	16	1	27	2	0.04
Elbow	2	54	10	2	27	1	0.06
Wrist/hand	6	46	14	3	26	1	0.09
Aggregated	8	35	23	5	21	4	0.02

• Algesia (needle prick) was assessed in five and the threshold to perception of vibration by use of a tuning fork 256 Hz in three innervation territories (Table 3) as formerly described [3]. Deviation of sensibility was classified as "severely reduced/changed" when an allodynic reaction was recorded, or when pain or vibration could either not be perceived at all or was altered sufficiently to be clearly apparent to the examiner from the patient's reaction. Deviation of sensibility was classified as "reduced/changed" with any other divergence from normal (dys-, hypo-, or hypersensibility). For the latter assessment, sensation was compared with sensibility in other territories assessed as normal (Table 1).

**Table 3:** Outcome of individual muscle testing at baseline and at follow-up. Analysis by Wilcoxon signed-rank test of the relation between findings at the two occasions (for subjects examined twice)

	**Intervention subjects. N = 69**					**Control subjects. N = 28**				
				
**Muscle**	**Number with weakness at baseline**		**Number with weakness at follow-up**		**P**	**Number with weakness at baseline**		**Number with weakness at follow-up**		**P**
				
	**Grade 4+**	**Grade 4 or less**	**Grade 4+**	**Grade 4 or less**		**Grade 4+**	**Grade 4 or less**	**Grade 4+**	**Grade 4 or less**	
Pectoral	0	0	0	0	-	0	0	0	0	-
Deltoid	9	3	9	0	0.23	8	1	1	0	0.005
Latissimus	3	0	1	0	0.15	5	0	1	0	0.10
Infraspinatus	6	0	3	0	0.18	2	0	1	0	0.32
Biceps	7	2	8	0	0.32	7	1	2	0	0.03
Triceps	24	1	20	0	0.20	12	1	11	0	0.37
Radial flexor of wrist	12	1	11	0	0.58	7	1	7	0	0.71
Short radial extensor of wrist	10	2	9	0	0.15	8	0	6	0	0.53
Ulnar extensor of wrist	18	1	11	0	0.08	7	0	10	0	0.37
Short abductor of thumb	18	1	12	0	< 0.05	7	1	8	0	0.94
Abductor of small finger	5	0	3	0	0.32	0	0	0	0	-

• The mechanosensitivity (soreness) of nerve trunks was examined at seven locations by palpating with a moderate manual pressure (3 kp) from proximal to distal (Table 4). Mechanical allodynia was quantified according to Table 1. "Severe" mechanical allodynia was registered with avoidance reaction/jump sign, "moderate" allodynia when the patient expressed the pressure as seriously uncomfortable and "mild" allodynia with the presence of any other soreness exceeding normal. For the latter assessment, the level of soreness was compared to reactions regarded as normal to pressure elsewhere along nerves (Table 1).

**Table 4:** Sensory findings at homonymously innervated territories at baseline and at follow-up. Analysis by Wilcoxon signed-rank test of the relation between sensibility (algesia and vibratory threshold) at the two occasions (for subjects examined twice)

		**Intervention subjects. N = 69**					**Control subjects. N = 28**				
					
**Innervation territory**		**Number of abnormalities at baseline**		**Number of abnormalities at follow-up**		**P**	**Number of abnormalities at baseline**		**Number of abnormalities at follow-up**		**P**
					
		**Slight**	**Severe**	**Slight**	**Severe**		**Slight**	**Severe**	**Slight**	**Severe**	
Algesia	Axillary	19	0	18	1	0.97	5	0	7	0	0.53
	Musculocutaneous	15	1	13	1	0.65	5	0	6	0	0.30
	Median	13	0	13	0	1.00	2	0	4	0	0.41
	Radial	13	0	8	0	0.17	5	0	4	0	0.71
	Ulnar	1	0	1	0	1.00	1	0	0	0	0.32

Vibration	Median	42	1	30	1	0.30	11	0	11	0	0.10
	Radial	46	0	27	0	0.005	20	0	10	0	0.17
	Ulnar	16	0	7	0	0.07	12	0	9	0	1.00

The examiner was aware of the affiliation of each examined subject to one or the other division of the company but was otherwise blinded to any information relating to the study subjects including their answers to the questionnaire. No communication occurred during the physical examination except for instructions from the examiner and reactions from the subjects to the applied tests.

### Intervention

A physiotherapist from the occupational health service instructed subjects in the intervention group in stretching exercises based on neurodynamic principles [24,25]. Groups of 10–12 employees were instructed in sessions of 20 minutes during which the exercises were demonstrated twice. A pamphlet with text and illustrations of the stretching exercises were handed out to the participants who were encouraged by the therapist to complete the program at least three times daily during work hours and additionally after hours over a 6-months period. During the intervention the therapist was available at the worksite once a month for employees who wanted ergonomic consultations. The intervention was based on the "intention to treat" concept and no further encouragement to continue stretching was provided.

**Figure 3:**
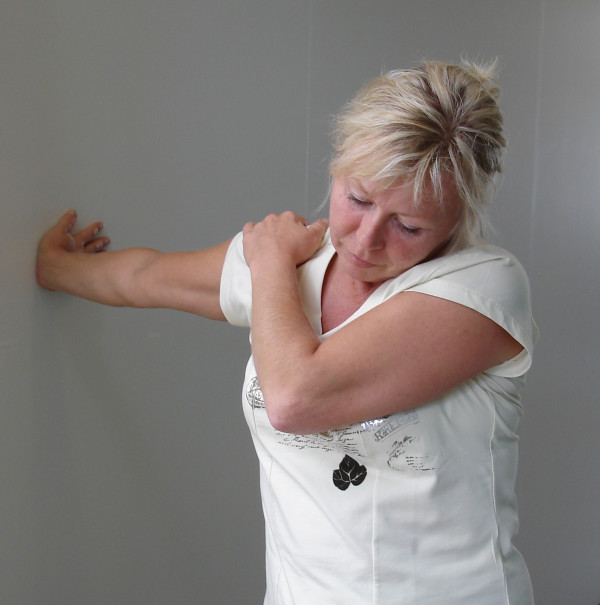
Stretching 3 addressing the structures surrounding the median nerve (right side).

**Figure 4:**
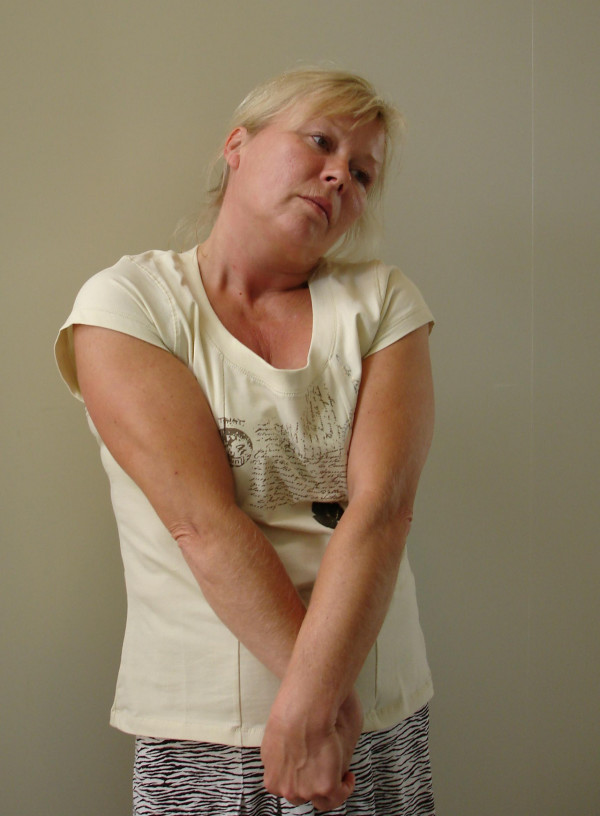
Stretching 4 addressing the structures surrounding the radial nerve (right side).

The first (Stretching 1) and second (Stretching 2) exercise aimed to stretch the volar forearm flexors and the second additionally to stretch the pronator muscle. The third (Stretching 3) and the fourth (Stretching 4) exercises, respectively, aimed to mobilize the median and radial nerve, respectively. The following instructions for stretching were given:

• Stretching 1: "Keep your arms along the front of the body with extended elbows. Fold your hands and rotate forearms to point the dorsum of the hands backwards. Raise your completely extended arms overhead and maximally backwards. Flex one elbow behind your neck while gripping the elbow with your opposite hand pulling it towards the middle. Keep this position for a few seconds. Repeat on the other side. Stretch arms and move them in the lateral direction and back to the start position. Repeat one time. Duration approximately 30 seconds".

• Stretching 2: "Place yourself standing at the side of your desk with extended elbows, outward-rotated forearms and fingers pointing backwards toward your body, palms flat on the desk and wrists extended maximally backwards. Repeat one time. Duration approximately 20 seconds".

• Stretching 3: "Place your hand flat on a wall with fingers pointing backwards, elbow stretched, and shoulder lowered (kept down by the other hand) and if possible flex your head away from the arm. Duration approximately 20 seconds. Repeat on the other side" (Figure 3).

• Stretching 4: "Place your thumb in the palm and grip around your thumb with maximal forearm inward-rotation. Grip hand/fingers with the opposite hand and flex the inward-rotated wrist. Lower the shoulders. Extend neck backwards away from arm. Duration approximately 20 seconds. Repeat on the other side" (Figure 4).

During the study period no other work organizational interventions had occurred at any of the two sites. Besides the physical examination the control group received neither any placebo intervention nor any other attention of any kind.

### Outcome data

The main outcomes were changes with regard to symptoms and physical findings. We have looked at the following data:

• Self-reported change of pain level. Calculations were made for subjects who answered the follow-up questionnaire.

• Changes from baseline to follow-up among subjects in the intervention group and among the controls

◦ of self-reported pain. Calculations were made for subjects who answered both questionnaires.

◦ of neurological findings in isolation and of their occurrence in patterns in accordance with the presence of afflictions of the brachial plexus, the posterior interosseous nerve, and the median nerve at elbow level, respectively. The definition of neurological patterns has been described previously [5]. Calculations were made for subjects who participated in both physical examinations.

• The development of pain in subjects with no pain or with a minor pain score (less than 2) summarized for three regions (hand, elbow, shoulder). Calculations were made for subjects who answered both questionnaires and participated in the first physical examination.

### Statistics

Paired samples were studied by a Wilcoxon signed rank-sum test and non-paired samples of the same parameters by a two-sample Wilcoxon rank-sum (Mann-Whitney) test.

All calculations were made by the STATA statistical packet ver. 8.2.

## Results

All the presented results refer to the right upper limb.

Compared to the intervention subjects the controls were slightly older (mean age 37 and 41, respectively) and the proportion of women higher (66% and 33%, respectively). The intervention subjects and the controls were comparable with regard to mean body mass index (25 and 24, respectively).

Compliance with the recommended intervention was generally good. Among the 66 subjects in the intervention group who answered the second questionnaire 60 affirmed that they had regularly completed the stretching exercises at the recommended rate and 53 that they included all exercises. Two subjects out of the 30 controls performed some sort of stretching (which would most likely differ from the recommended exercises). The content of the work, work hours and ergonomic features of work sites were unchanged and comparable in the two groups during the course of the intervention.

At baseline/follow-up 120/66 computer operators in the division in Esbjerg and 44/30 in Aarhus, respectively, answered questionnaires about upper limb symptoms and 80/70 computer operators in Esbjerg and 37/28 in Aarhus, respectively, were subjected to physical examinations by the same examiner (JRJ) (Figures 1, 2).

### Symptoms

The baseline pain level was identical in the intervention group and the control group. In the mouse-operating limb pain was experienced by 67 subjects with summarized pain being mostly slight (median score = 2, range 0 – 16) on a VAS scale 0 – 29. Contralateral pain was present in 24 subjects and of lower intensity (median score = 0, range 0 – 14). The summarized score exceeded 4 in 33 and 13 limbs, respectively, on the two sides [5].

**Figure 5:**
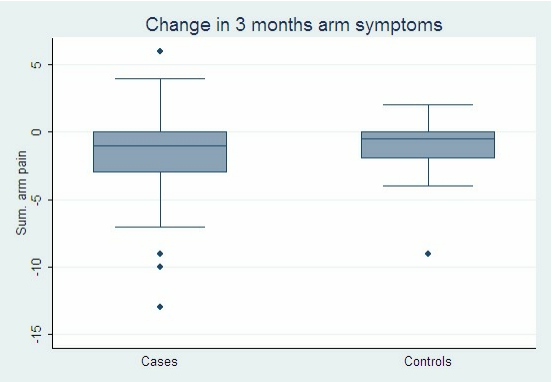
The summarized pain score in the intervention group (64 persons) and the control group (18 persons) before and after the intervention.

On follow up after six months the application of the Wilcoxon signed-rank test showed a significantly reduced pain level among the 64 subjects in the intervention group who answered both questionnaires (z = -3.368, p = 0.0008). No statistical change could be demonstrated for the 18 controls (z = -1.590, p = 0.12) (Figures 1, 2 and 5).

Application of a two-sample Wilcoxon rank-sum test (Mann-Whitney) was unable to demonstrate a significant difference between the intervention group and the control group (z = – 0.745, p = 0.46).

However, following the intervention, 23 out of 66 subjects in the intervention group who answered the second questionnaire reported improvement and 8 reported more pain than before. In the control group the perceived changes in pain in each direction were almost equal as 4 out of 30 subjects reporting fewer symptoms while 5 reported increased pain (Figures 1, 2).

A significant improvement was noted for the shoulder per se (p = 0.04) but no significant change was reached for the elbow and wrist/hand. Aggregation of data on symptoms in the three regions resulted in an overall significant improvement during the intervention (p = 0.02) (Table 2).

### Findings

#### Individual findings

The changes relating to each physical parameter from baseline to follow-up for the 69 subjects in the intervention group and the 28 controls who were examined twice is illustrated in Tables 3, 4, 5 (Figures 1, 2). A significant improvement with regard to muscle function was reached in the intervention group for the short abductor of the thumb muscle and in the control group for the deltoid and biceps muscles (Table 3). Algesia was not changed for any innervation territory while the vibratory sense improved significantly for the radial nerve in the intervention group (Table 4). Mechanosensitivity was significantly improved in the intervention group for the median nerve (elbow) and the posterior interosseous nerve, and in the control group for the infraclavicular portion of the brachial plexus (Table 5).

**Table 5:** Mechanosensitivity of nerve trunks at baseline and at follow-up. Analysis by Wilcoxon signed-rank test of the relation between mechanosensitivity at the two occasions (for subjects examined twice)

	**Intervention subjects. N = 69**					**Control subjects. N = 28**				
				
**Mechanosensitivity of nerve trunks**	**Number with mechanical allodynia at baseline**		**Number with mechanical allodynia at follow-up**		**P**	**Number with mechanical allodynia at baseline**		**Number with mechanical allodynia at follow-up**		**P**
				
	**Mild**	**Moderate to severe**	**Mild**	**Moderate to severe**		**Mild**	**Moderate to severe**	**Mild**	**Moderate to severe**	
Supraclavicular brachial plexus	3	0	0	0	0.08	2	0	0	0	0.16
Clavicular brachial plexus	3	1	3	0	0.41	3	0	2	0	0.65
Infraclavicular brachial plexus	14	6	14	0	0.05	8	5	4	0	0.004
Median nerve (elbow)	18	3	8	0	0.0003	8	5	6	0	0.002
Posterior interosseous nerve	26	4	13	0	0.0001	13	3	9	0	0.03
Ulnar nerve (sulcus)	0	0	0	0	-	1	0	0	0	0.32
Median nerve (carpal tunnel)	0	0	1	0	0.32	1	0	0	0	0.32

#### Summarized individual findings

The summarized individual physical findings was reduced in 35 and increased in 18 out of the 69 subjects in the intervention group and in 13 and 10, respectively, of the 28 controls that were physically examined twice (Figures 1, 2). For the entire sample, the application of a Wilcoxon signed-rank test demonstrated a significant reduction of physical findings at follow-up (p = 0.005). However, a two-sample Wilcoxon rank-sum (Mann-Whitney) test could not demonstrate any difference between the two groups (p = 0.86) thus suggesting that physical findings were unaffected by the intervention.

#### Patterns

According to the criteria for definition of the three patterns of physical findings reflecting nerve-afflictions there was a tendency towards fewer patterns in both groups, but particularly in the intervention group in which 29 patterns were identified at baseline and 7 at follow-up. Among the controls the corresponding numbers were 18 and 9, respectively. This apparent difference was not statistically significant.

#### Incident symptoms and findings

For subjects scoring less than two on pain at the first measurement (meaning minimal pain) we have analysed the change at follow-up of summarized pain score of at least one for 15, 12, and 14 limbs, respectively, with a pattern in accordance with brachial plexopathy, posterior interosseous neuropathy, and median neuropathy at elbow level, respectively. The slopes of the regression lines turned out to be significantly positive for the patterns illustrating the brachial plexus (p = 0.005) and the posterior interosseous nerve (p = 0.02) and borderline significant for the pattern reflecting the median nerve at elbow level (p = 0.052). In spite of few numbers these findings suggest the relation of incident symptoms to pathology at the three levels.

## Discussion

Any preventive intervention should preferably be based on an understanding of the phenomena underlying the disorder or at least a theory founded on evidence with regard to this issue. In the absence of such understanding, any intervention may target irrelevant issues and would most likely be ineffective. The intervention in this study was based on indications from a former study of the relation of symptoms to upper limb nerve afflictions at three locations (brachial plexus at chord level, posterior interosseous nerve and median nerve at elbow level) [5]. Accordingly, a six month course of stretching was designed with the aim to mobilize the nerve segments at these locations.

The ability of the intervention to reduce symptoms in the studied sample of computer operators is encouraging. The decrease after the intervention of physical findings in the entire sample could not be related to the stretching exercises, however. This is not necessarily contrary to the favourable subjective improvement, but may be explained by statistical weaknesses and other potential sources of error.

First of all, the physical work environment of this small sample of computer operators was already optimized prior to the study. They also had fewer symptoms than reported in other studies on upper limb complaints in computer workers. These favourable circumstances in terms of health would tend to make it more difficult to demonstrate an effect of the intervention.

Secondly, 20 computer workers did not answer the questionnaire at start and 88 did not participate at the follow-up (Figures 1, 2). For the physical examinations the corresponding figures were 67 and 31 subjects, respectively. In fact only 62 subjects in the intervention group and 16 controls participated in all parts of the study. This small number especially of controls is a clear weakness, which together with the high number of limbs without symptoms and findings reduces the statistical power of the study.

The composition of the intervention group and the control group was comparable with respect to gender and age. We did not analyse for prior disorders and psychosocial factors but the two groups were of similar composition with respect to age, sex, and educational and social background. We consider bias due to differences in exposure or vulnerability in the two groups unlikely because of the almost identical content and organization of work, workstation ergonomics and psychosocial work environment. We did not analyse for other covariates such as prior disorders and psychosocial factors but the two groups were comparable with respect to these factors.

Complete blinding of the physical examination could not be achieved. This would demand randomization to the intervention which, however, was deliberately offered to staff in one division of an engineering company with controls in a geographically separated division in order to avoid mutual contacts between intervention and control subjects and thus to prevent the controls to also engage in the stretching exercises if the intervention subjects would perceive them as beneficial. Apart from this, subjectivity was reduced by performing all physical assessments blinded to any other information about the studied computer operators.

Findings were entered into patterns according to predefined algorithms. Still, it cannot be excluded that one finding, e.g. of weakness in a specific muscle, can bias other findings such as sensory deviations because all physical examinations were made by the same examiner. The execution of the examination may have changed slightly from baseline to follow-up. However, the intra-examiner reliability of the applied examination is likely to be good because the inter-examiner reliability has previously been found satisfactory [2,3]. It may also be argued that a tendency of the study subjects towards more familiarity with the physical examination at the second examination could result in muscle function for example improving over time or nerve trunk soreness being perceived as less uncomfortable than previously. These potential sources of bias cannot be overcome.

A certain fluctuation of findings was noted in the intervention group as well as among controls. However, persistence for some time of physical neurological findings which appear to be characteristic for peripheral nerve afflictions would result in a reduced influence on physical findings of the intervention. Our clinical experiences with assumed neuropathic upper limb conditions suggest that patients reporting fewer symptoms at control visits after successful treatment may still present unchanged levels of pareses and sensory dysfunction. It therefore seems possible that a significant reduction of physical findings would require a follow-up of longer duration.

There is no indication that effective prevention can be accomplished by the isolated use of stretching exercises. However, as a supplement to ergonomic and organizational change stretching may well contribute to reduce the burden of computer-related upper limb disorders.

The outcome of this intervention may serve to further illustrate the character of computer-related upper limb disorder by indicating the relation of incident symptoms to neurological patterns suggesting nerve-afflictions with specific locations. Consequently, it contributes to a further validation of the employed physical examination [2-4].

## Conclusion

When performed three times a day stretching designed to improve the available space and mobility of nerves at the infraclavicular brachial plexus and the posterior interosseous and median nerves at elbow level can reduce computer-related upper limb pain. However, no firm conclusions can be drawn with regard to an influence on physical findings except for the observation that incident symptoms were related to findings suggesting pathology at the three levels. The results of this study may contribute to the prevention of computer-related upper limb disorders and also provide support to hypotheses on the role of peripheral nerve-afflictions in these conditions.

## Competing interests

The author(s) declare that they have no competing interests.

## Authors' contributions

G Thomsen and J R Jepsen have designed the study. J R Jepsen collected the data relating to the physical examination. G Thomsen was responsible for the electronic questionnaire, data-management and the statistical calculations. J R Jepsen prepared the manuscript which has been approved by G Thomsen.
